# Multifunctional Nanomaterials for Biofortification and Protection of Tomato Plants

**DOI:** 10.1021/acs.est.3c02559

**Published:** 2023-09-27

**Authors:** Belén Parra-Torrejón, Andrés Cáceres, Manu Sánchez, Luis Sainz, Miguel Guzmán, Francisco J. Bermúdez-Perez, Gloria B. Ramírez-Rodríguez, José M. Delgado-López

**Affiliations:** † Department of Inorganic Chemistry, Faculty of Science, University of Granada, Av. de Fuente Nueva, s/n, 18071 Granada, Spain; ‡ Institute of Nanoscience and Materials of Aragon, INMA-CSIC, C/Mariano Esquillor, s/n, 50018 Zaragoza, Spain; § Department of Agronomy, University of Almeria (RNM 151 PAIDI-UAL, ceiA3, CIAMBITAL), Carr. Sacramento, s/n, La Cañada, 04120 Almería, Spain; ∥ Nanointec S.L. Pol. Ind. de la Celulosa, C. Regaliz, 6, 04007 Almería, Spain

**Keywords:** nanoparticles, calcium phosphate, zinc, nanofertilizer, antibacterial, agriculture, biofortification, tomato

## Abstract

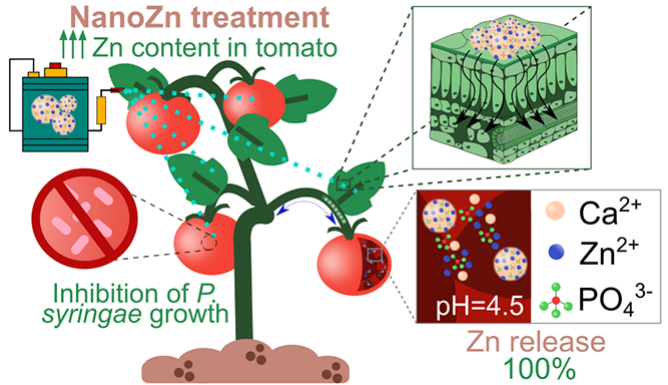

Calcium phosphate nanoparticles were doped with zinc ions to produce multifunctional nanomaterials for efficient agronomic fortification and protection of plants. The resulting round-shaped nanoparticles (nanoZn) were composed of 20.3 wt % Ca, 14.8 wt % P, and 13.4 wt % Zn and showed a pH-controlled solubility. NanoZn were stable in aqueous solutions at neutral pH but dissolved in citric acid at pH 4.5 (i.e., the pH inside tomato fruits), producing a pH-responsive delivery of the essential nutrients Ca, P, and Zn. In fact, the foliar application of nanoZn on tomato plants provided tomatoes with the highest Zn, Ca, and P contents (causing, respectively, a 65, 65, and 15% increase with respect to a conventional treatment with ZnSO_4_) and the highest yields. Additionally, nanoZn (100 ppm of Zn) inhibited *in vitro* the growth of *Pseudomonas syringae* (*Ps*), the main cause of bacterial speck, and significantly reduced *Ps* incidence and mortality in tomato seeds, previously inoculated with the pathogen. Therefore, nanoZn present dual agricultural applicability, enriching crops with nutrients with important metabolic functions in humans and simultaneously protecting the plants against important bacterial-based diseases, with considerable negative impact in crop production.

## Abstract 2

Improving human and environmental health through nanoenabled agriculture: Doping nanoscale calcium phosphate with Zn to efficiently enrich crops in essential macro- and micronutrients and simultaneously protect the plants from bacterial-based diseases.

## 1 Introduction

Agriculture needs to face the general challenge of the inefficient use of agrochemicals and the compelling environmental aspects derived therefrom.^1,2^ Biofortification (i.e., enriching the edible parts of the plants in micronutrients and macronutrients essential for human growth and health^3^) through soil fertilization is usually inefficient and expensive since the metallic ions are immobilized in the soil through precipitation, and so unavailable to plant roots.^4^ Foliar application of soluble metallic salts can overcome this issue but it can cause leaf burning due to the high dosage required to achieve an effective fortification.^5,6^ Conversely, foliar spray application of nanoenabled agrochemicals offers a unique opportunity to improve the nutrient use efficiency while reducing environmental pollution and promoting food security.^2,7−9^

Among the most promising nanomaterials, calcium phosphate nanoparticles (CaP-NPs), with similar physicochemical properties to the inorganic nanostructure of bone,^10,11^ are of special interest because their structure and chemical composition can be manipulated for a wide variety of functionalities. CaP-NPs can serve as a nutrient supplier due to their composition, mainly Ca and P, important macronutrients for plant and humans and of interest in agronomic biofortification.^3^ This along with the pH-dependent solubility, which allows a controlled release of the ionic constituents at slightly acidic pHs, makes these inorganic nanoparticles the ideal nanoplatform for the efficient nutrient fortification.^12,13^

The engineering of these materials can provide them with a wider degree of applicability and functionalities. CaP-NPs have high capacity to host foreign ions and/or adsorb active molecules to a high extent on the highly reactive surface.^14^ The incorporation of zinc (Zn), which is an essential micronutrient playing fundamental biological functions in plants and humans (e.g., cell growth and differentiation, DNA replication, gene expression, wound healing, and supporting a healthy immune system),^15^ can result in multinutrient nanomaterials for the agronomic fortification of fruits. Zn deficiency is among the most important factors causing illness and disease.^16^ According to the World Health Organization (WHO), Zn deficiency kills 430,000 children annually, becoming an important form of malnutrition worldwide.^17^ The regions where Zn deficiency in human beings is widespread present the highest percentages of potentially Zn-deficient soils or soils with low Zn availability (i.e., Iraq, Turkey, and Pakistan).^4^

To address this serious problem, the agronomic Zn biofortification has been explored to increase the Zn concentrations in grains or fruits.^15,16,18^ Foliar application of conventional soluble forms (e.g., zinc sulfate and EDTA-Zn chelates) can cause leaf burning, so several applications at low dosages may be required to correct the Zn deficiency.^5,6^ On the other hand, some studies indicated that the foliar application of zinc oxide nanoparticles (ZnO-NPs) seems to positively influence growth and yields of several plants (e.g., soybean, tomato, alfalfa, cucumber, peanuts, and green peas)^6,9^ and increase the Zn content in several fruits and grains;^19−23^ however, variable results between experiments and crops have been reported.^8^ In addition, ZnO-NPs can negatively affect plant growth and metabolism at different stages of development.^9,24^ ZnO-NPs can have other potential adverse effects, including toxicity to nontarget organisms, accumulation in the food chain, and environmental pollution.^25,26^ Therefore, nontoxic nanosystems for safer and more efficient agronomic fortification are required.

Interestingly, zinc ions (Zn^2+^) exhibit antimicrobial activity against various fungal and bacterial strains, including some relevant vegetal pathogens such as *Pseudomonas syringae* (*Ps*), the main cause of bacterial speck, one of the most common diseases of tomatoes.^27^ This serious bacterial disease appears in a form of small, sunken, and black lesions on tomato leaves, stems, and fruits, causing severe losses in tomato production (up to 75%) and quality worldwide.^28^ Bacterial speck occurs particularly during cold and wet springs, and its control remains difficult and limited to harmful pesticides.^29^

In this work, calcium phosphate nanoparticles have been doped with Zn ions to obtain multinutrient nanofertilizers (nanoZn) for the efficient agronomic fortification of plants and simultaneously protect the plants against vegetal pathogens. The resulting nanoparticles were characterized in depth in terms of size, structure, and chemical composition as well as stability in different media of practical interest. We evaluated the efficiency of nanoZn for the fortification of tomatoes (*Lycopersicon escullentum* Mill.), the second most important vegetable crop after potatoes, with approximately 186.8 million tons of tomato fruits produced in 2020.^30^ Considering that Zn has also been shown to induce disease resistance in plants and possess antibacterial activity,^27^ we also studied the protection effect of nanoZn against *Ps*. These nanomaterials thus offer great opportunities to increase plant nutritional values of tomato and crop yields and simultaneously protect the plant against vegetal pathogens. These multifunctional nanomaterials open the door toward new strategies to reduce the usage of fertilizers and pesticides and thus mitigate their negative environmental impact.

## 2 Materials and Methods

### 2.1 Materials

Analytical-grade reagents were purchased from Sigma-Aldrich: potassium citrate tribasic dihydrate (K_3_(C_6_H_5_O_7_)·2H_2_O, ≥99.0% pure), potassium phosphate dibasic anhydrous (K_2_HPO_4_, ≥99.0% pure), potassium hydroxide (KOH 85% pellet for analysis), calcium chloride dihydrate (CaCl_2_·2H_2_O, ≥99.0% pure), and zinc chloride (ZnCl_2_, ≥97% ACS reagent). Ultrapure water (0.22 μS, 25 °C, Milli-Q, Millipore) was used to prepare all of the solutions.

### 2.2 Synthesis and Characterization of NanoZn

Amorphous calcium phosphate (ACP) nanoparticles were synthesized through a clean, green, and scalable synthetic route inspired in bone mineralization.^10^ Two solutions of equal volume (100 mL) were mixed: (A) an aqueous solution containing CaCl_2_ (0.2 M) and K_3_C_6_H_5_O_7_ (0.2 M) and (B) an aqueous solution containing K_2_HPO_4_ (0.12 M) with pH around 12 (adjusted with KOH). After stirring for 5 min at room temperature, the samples were centrifuged (5000 rpm, 10 min) to collect the nanoparticles, washed twice with ultrapure water (5000 rpm, 10 min), and then stored at 4 °C. A small quantity of the sample was frozen at −20 °C and freeze-dried (Telstar) for further characterization. The same protocol was followed for the preparation of zinc-doped amorphous calcium phosphate nanoparticles (nanoZn) by adding ZnCl_2_ (40 mM) to solution A at an initial molar Zn/Ca ratio of 0.2.

Fourier transform infrared (FTIR) spectra of the samples were recorded on a Tensor 27 spectrometer (Bruker, Karlsruhe, Germany). Powdered samples (2 mg) were mixed with 200 mg of anhydrous potassium bromide (KBr), set into a 12 mm diameter disc, and pressed at 5 tons in a hydraulic press (Specac). Three pellets were produced for each sample, and a KBr pellet without sample was used as blank. The infrared spectra were recorded from 400 to 4000 cm^–1^ at a resolution of 4 cm^–1^. X-ray powder diffractograms (XRPD) were recorded on a Bruker AXS D8 Advance diffractometer using Cu Kα radiation (λ = 1.5418 Å), from 8 to 60° (2θ) with a scan rate of 0.5 s/step and a step size of 0.02° with an HV generator set at 40 kV and 40 mA. High-angle annular dark field-scanning transmission electron microscopy (HAADF-STEM) images, selected area electron diffraction (SAED) patterns, and energy-dispersive X-ray (EDS) spectra of nanoZn were acquired with an STEM FEI TALOS F200X microscope equipped with 4 Super-X SDDs (Thermo Fisher Scientific Waltham, MA) of CIC-UGR. To this purpose, nanoZn nanoparticles were ultrasonically dispersed in ultrapure water, and then, some drops of the slurry were deposited on 200 mesh copper grids covered with thin amorphous carbon films. Nanoparticle chemical composition was evaluated by inductively coupled plasma optical emission spectroscopy (ICP-OES, Optima 8300, PerkinElmer, from CIC-UGR). To this aim, 10 mg of the powdered samples was dissolved in 1.5 mL of ultrapure nitric acid, and then, the mix was made up to 50 mL with ultrapure water. The samples were measured in triplicate at their correspondent emission wavelengths: 317.93 nm (Ca), 213.62 nm (P), and 206.20 nm (Zn). The surface charge of the nanoparticles (ζ, mV) was measured by dynamic light scattering with a Litesizer 500 (Anton Paar, Austria).

The quality assurance (QA) and quality control (QC) operations carried out to establish the methods for sample quantifications and to validate the analytical instruments (ICP-OES) are described in Section S1 of the Supporting Information.

### 2.3 Chemical and Structural Stability of the Nanoparticles

#### 2.3.1 Long-Term Stability under Storage Conditions

NanoZn suspension (10 wt % in water) was stored at 4 °C. After 2 years, 2 g of the sample was washed two times with ultrapure water by centrifugation (5000 rpm, 10 min) to eliminate the released ions and freeze-dried overnight. Then, the powdered samples were characterized by ICP-OES to quantify the Zn content and XRD to evaluate whether ACP converts into a more stable calcium phosphate phase.

#### 2.3.2 Stability in Diluted Aqueous Media

Zn^2+^ release from nanoZn was evaluated in ultrapure water at neutral pH and in a citric acid solution (10 mM) with the pH adjusted to 4.5. This latter media mimics the conditions inside the tomato fruit.^31^ To this aim, 25 mg of nanoZn was immersed in 25 mL of each solution (water and citric acid solution) to reach a Zn^2+^ concentration of 100 ppm. Three replicates were prepared for each condition. After 24 h of incubation at room temperature, the respective samples were centrifuged at high speed (12000 rpm for 2 min). Then, centrifugal filtration of the supernantant (Vivaspin 6, MWCO 10 kDa, 8000 rpm, 10 min) was carried out to remove dispersed nanoparticles. To analyze the released ions by ICP-OES, 20 mL of the supernatant of each replicate was treated with 750 μL of nitric acid (65%) and diluted up to a final volume of 25 mL in a volumetric flask (dilution factor 1.25). The emission wavelengths were 206.20 nm (Zn), 317.93 nm (Ca), and 213.62 nm (P). We calculated the weight percent of the released ions (Ca, P, or Zn) with respect to the initial mass of the corresponding element in the nanoparticle, as follows:
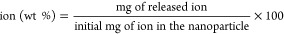
1

### 2.4 Experimental Design for Plant Growth and Biofortification

#### 2.4.1 Plant Growth Conditions

Plantlets of commercial tomato cherry (*Lycopersicon esculentum* Mill. var. *cerasiforme*, cv. HTL1708480, Axia Vegetable Seeds) were transplanted at 35 days post sowing (dps) in a greenhouse at Universidad de Almeria (36°49′45″N 2°24′16′′W) during the summer of 2018 under hydroponic conditions.

The environmental conditions (temperature of 27 °C and humidity of 65%) were managed by mechanical ventilation and a traditional bleaching system. The irrigation supply was 40 mL, and the frequency was determined by the balance between supply and drainage in a range of 7–18 irrigations per day. An automatic system (Himarcan R01A) was used to supply a basic nutrient solution with the following composition: 11 mM N (10 mM NO_3_^–^ and 1 mM NH_4_^+^); 1 mM P; 8 mM K; 2 mM Ca; 1.5 mM Mg; 2 mM S; 4 μM Fe; 2 μM Mn; 1 μM Zn; 1 μM Cu; 1 μM B; and 0.5 μM Mo. The nutrient solution was applied to all the crops (treated and nontreated plants) to supply enough nutrients for growing plant without soil, under hydroponic conditions.^32^ The electrical conductivity of irrigation water was 1.71 dS/m and the pH = 5.5.

#### 2.4.2 Foliar Application of Zn-Based Treatments for Fruit Fortification

Approximately 90 dps, at the first stage of fruit development (i.e., fruit setting stage), tomato plants received two different foliar treatments: (i) nanoZn and (ii) zinc sulfate. The compounds were dispersed (nanoparticles) or dissolved (ZnSO_4_) in deionized water (total Zn concentration = 100 ppm) with a magnetic stirrer (ANZESER SH-2) for 30 min. The resulting aqueous solutions were directly sprayed onto the leaves with an atomizer. Some fruits were also irremediably sprayed. Each plant (15 replicas per treatment) was sprayed with 500 mL of the corresponding solution. The sprayed solution remained on the leaves and on some of the fruits but did not runoff into the soil. Each application was repeated 4 times, one per week, for a month. Control plants (*n* = 15) were sprayed with deionized water with the same volume and frequencies.

#### 2.4.3 Fruit Harvesting and Sample Analysis

Healthy tomato fruits were hand harvested from each plant when they reached the mature red-ripe stage (color classification number 6, i.e., more than 90% of the surface is red, according to the United States Standards for Grades of Fresh Tomatoes).^33^ Color was recorded with a Chromameter CR-400 (Konica Minolta). The total number of fruits and total fruit weight were measured from 15 different plants. Phenol contents of extracts from 10 different fruits were determined with Folin-Ciocalteu’s assay, according the experimental procedure described in ref (34). Total soluble solids (TSS) were determined by using a digital refractometer PR-32 (ATAGO KERM). Several drops of tomato juice of each fruit were deposited on the prism of the refractometer, with the result being expressed as °Brix. Ten fruits from different plants were analyzed.

Prior to the quantification of the nutrient content (Zn, Ca, P, and N), tomatoes were repeatedly and thoroughly rinsed, first with tap water to remove dust and impurities and then with ultrapure water (1 L per fruit cluster) to prevent the accumulation of residual nanoparticles or salts on the fruit surface. Each tomato was then cut in 24 pieces, and one to two pieces for each tomato were selected to obtain 31 g of “representative sample” per treatment. The samples were dried at 70 °C for 72 h in an oven. Following the Kjeldahl procedure, a dried sample of 0.25 g was passed through a Kjeldahl flask for a wet digestion with H_2_SO_4_/H_2_O_2_ to determine the nutrient content. To evaluate the nitrogen content, 1 mL of mineralized sample and 25–30 mL of NaOH were mixed in the Kjeldahl flask and then connected to the Bouat device until the production of ammonia vapor. The latter was directed to the collecting flask to be evaluated with Shiro-Tashiro dye. To determine the phosphorus content, the mineralized samples were placed into an Erlenmeyer volumetric flask and mixed with a solution previously prepared with ammonium molybdate, potassium antimony tartrate, and l-ascorbic acid. The phosphorus content was determined by a spectrophotometer at 700 nm following the protocol of phosphomolybdic complexes.^35^ Ca and Zn were analyzed using inductively coupled plasma mass spectrometry (ICP-MS, XSERIES 2, Thermo Fisher, Research Facilities of Universidad de Almería, UAL). Average values of element composition in the tomato fruit were expressed as the mass (mg) of the element per 100 g of fruit fresh weight (mg/g FW).

### 2.5 Protection against *Ps*

The pathogenic strain of *P. syringae* (*Ps*, CECT 126) was purchased at the Colección Española de Cultivos Tipo (CECT). The bacteria were incubated in tryptic soy broth (TSB, No. 2, Sigma-Aldrich) at 26 °C overnight, following the supplier recommendations.

The inhibitory activity of nanoZn was evaluated by monitoring the absorbance (λ = 600 nm) of *Ps* in King’s B^36^ (KB, adjusted to pH = 4.5 with HCl) media at 30 °C using 96-well plates in a NanoQuant microplate reader (Thermo Fisher Scientific). The following treatments were carried out: (i) bacterial control (*Ps*), (ii) *Ps* in the presence of ZnSO_4_ at 100 ppm of Zn (*Ps* + ZnSO_4_), (iii) *Ps* in the presence of nanoZn at 100 ppm of Zn (*Ps* + nanoZn), and (iv) *Ps* in the presence of ACP (*Ps* + ACP). The conditions are summarized in Table SI1. A stock bacterial suspension was prepared by adding viable bacteria in King’s B medium to ensure a final optical density (OD) close to 0.1. Blank curves were obtained for each condition replacing 20 μL of stock bacterial suspension in King’s B by 20 μL of King’s B media. The absorbance of the blanks (nearly zero) was subtracted to the absorbance of the bacterial growth curves, the resulting data being represented as optical density (OD). Each condition was determined in triplicate.

Antibacterial effect of nanoZn against bacterial speck of tomatoes was also evaluated in tomato plants inoculated with *Ps*.^37^*Ps* (CECT 126) were grown in European Bacteriological Agar at 30 °C for 48 h. Then, the culture of *Ps* was collected in saline solution at a concentration of 2 × 10^7^ CFU/mL. Beefsteak tomato seeds (TOM21002) of “Marmande” type, susceptible to *Ps*, were obtained from Seeds for Innovation S.L company (Spain). Tomato seeds were sterilized previously by washing respectively with 20% sodium hypochlorite aqueous solution followed by three rounds of sterile distilled water. The seeds were germinated in Petri dishes neat at 20 °C in the darkness. After 5 days of germination, the seedlings with 3–4 cm long hypocotyls were completely submerged in 20 mL of bacterial suspension (2 × 10^7^ CFU/mL) and exposed to the bacterial suspension for 5 min with gentle mixing in an automatic stirrer at 100 rpm. Then, the inoculum was discarded, and the tomato seedlings were incubated in a growth room at 22 °C with a 12 h-dark photoperiod. Two different Zn sources were added to the inoculated seedlings: Treatment 1 (nanoZn, 100 ppm of Zn) and Treatment 2 (ZnSO_4_, 100 ppm Zn). Another set of seedlings were used as negative control C– (no inoculated + sterile water) and positive control C+ (inoculated + sterile water). Ten seedlings were evaluated per Petri dish, and three replicates were assigned to each treatment and controls.

The assays were monitored for 10–12 days post inoculation (dpi) to evaluate the symptom development and quantify the pathogen incidence and the mortality ratio.

### 2.6 Statistical Analysis

Statistical comparisons were analyzed with GraphPad Prism software (version 6.0) using one-way analysis of variance (ANOVA) and Bonferroni’s post hoc and *t*-Student unpaired tests. When *p*-values are lower than 0.05 (i.e., *p* < 0.05), differences in the obtained numerical results were considered statistically significant.

## 3 Results and Discussion

### 3.1 Synthesis and Characterization of NanoZn

**Figure 1:**
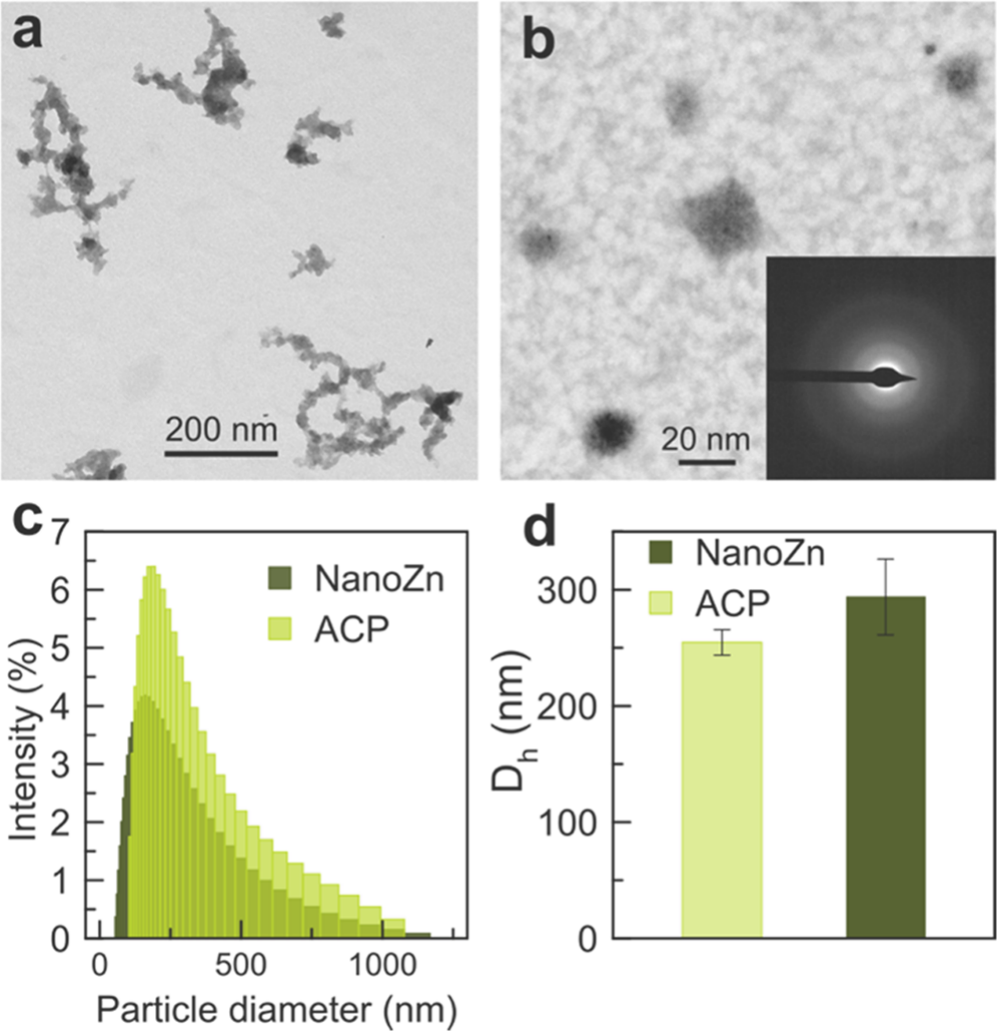
TEM micrographs of nanoZn with the typical rounded morphology of ACP (a, b). Panel (b) shows individual nanoparticles with an average diameter of 25 ± 2.6 nm (*n* = 50). SAED (inset) reveals a diffuse ring characteristic of nanoparticles of an amorphous nature. (c) Particle diameter distribution of nanoZn and ACP (control) in aqueous media as obtained by DLS (*n* = 10). Panel (d) shows the corresponding hydrodynamic diameter and polydispersity index (PDI, as error bars), which remains stable in time.

NanoZn are composed of round-shaped nanoparticles with an average diameter of 25 ± 2.6 nm (Figure 1a,b). They exhibit a similar morphology and size to nondoped amorphous calcium phosphate nanoparticles (Figure SI1).^38^ In fact, XRD and FTIR results confirmed that nanoZn are nonordered (amorphous) calcium phosphate similar to those obtained without Zn doping (Figure SI2). The TEM micrograph of nanoZn also revealed the existence of interactions between nanoparticles forming larger aggregates of around 250 nm in diameter. The formation of these aggregates likely occurs in solution, resulting in the average particle diameter (the hydrodynamic diameter, *D*_h_) observed by DLS (Figure 1c). It is important to note that this value of *D*_h_ remains constant in time. In general, Zn doping does not seem to affect the nanoparticles in terms of structure, size, or morphology.

**Figure 2:**
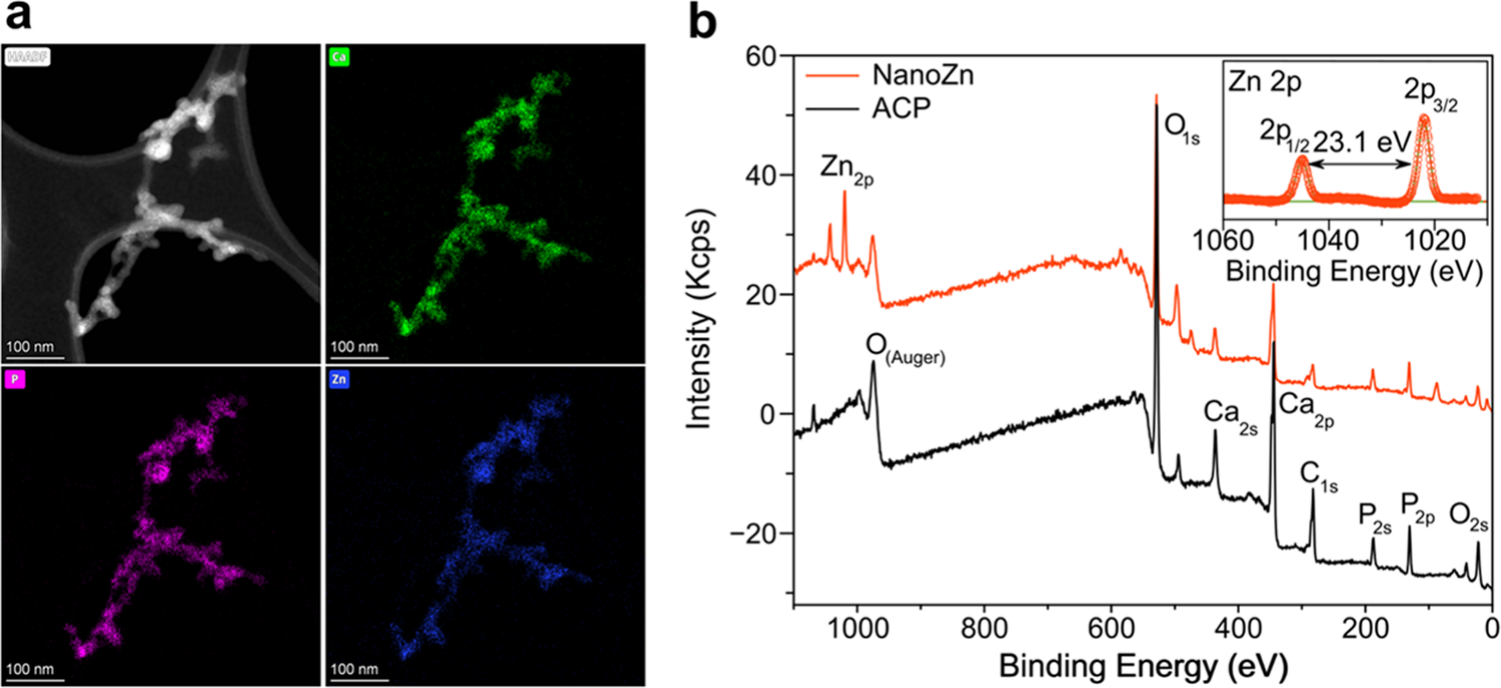
(a) HAADF-STEM micrograph of nanoZn and corresponding EDS maps showing nanoparticle composition: Ca (green), P (pink), and Zn (blue). (b) XPS survey spectra of nanoZn and ACP. The inset shows the high-resolution spectra of nanoZn in the Zn 2p spectral region showing two peaks due to spin–orbital splitting: 2p_1/2_ and 2p_3/2_.

Atomic-scale EDS chemical maps of individual nanoparticles indicated that Zn (blue) was homogeneously distributed along with Ca (green) and P (pink), as the main atomic components (Figure 2a). This confirmed the homogeneous incorporation of Zn into the calcium phosphate nanoparticles. The chemical composition of the nanoparticles was further studied by X-ray photoelectron spectroscopy (XPS). The survey XPS spectra (Figure 2b) indicated that doped and nondoped samples are composed of Ca, P, and O (in addition to remaining Na, peak at ca. 1070 eV). High-resolution XPS spectra of O 1s, Ca 2p, and P 2p levels of both samples were very similar to no significant differences in peak positions and in accordance with the formation of amorphous structures.^39^ The Zn 2p spectral line results in a doublet due to spin–orbital splitting with a separation of 23.1 eV between the 2p_1/2_ and 2p_3/2_ components (inset of Figure 2b), suggesting that Zn atoms present an oxidation state of +2, most probably interacting with the O of the tetrahedral PO_4_ groups.

These results were complemented by the quantification of the chemical composition of the whole sample by ICP-OES (Table 1). The content of Zn accounted for 13.4 ± 0.1 wt %. The incorporation of Zn^2+^ ions prompted to a slight decrease of Ca content likely due to the partial substitution of Ca^2+^ by Zn^2+^ ions in the ACP structure.^40^ The high Zn content can be associated with the high capacity of ACP to host a large variety of cationic and anionic substitutions, much higher than that of crystalline apatite (HA).^38^ The decrease in the metal/P ratio was mainly associated with an increase in the P content when Zn is incorporated. We hypothesize that Zn incorporation could induce variations in the surface functional groups, i.e., the incorporation of Zn causing an increase of the surface concentration of phosphate groups. However, the adsorption of part of the Zn^2+^ ions on the surface of the nanoparticles cannot be excluded. These surface modifications could indeed explain the decrease in the ζ-potential observed for nanoZn in comparison to ACP nanoparticles (Table 1).

**Table 1:** Chemical Composition and ζ-Potential of Fresh Samples (ACP and NanoZn) and the Samples Stored for 2 Years (NanoZn(St))^a^

sample	Ca (wt %)	P (wt %)	Zn (wt %)	(Ca + Zn)/P (molar ratio)	ζ (mV)
ACP	25.2 ± 0.6	12.3 ± 0.3		1.58 ± 0.01	–13.3 ± 2.7
NanoZn	20.3 ± 0.1***	14.8 ± 0.2***	13.4 ± 0.1***	1.49 ± 0.02***	–19.6. ± 1.7*
NanoZn(St)	20.3 ± 0.2	13.9 ± 0.1**	12.1 ± 0.1***	1.54 ± 0.01*	–18.2 ± 0.2

a Data are expressed as mean ± standard deviation (*n* = 3). Statistical analysis using the one-way ANOVA test and Bonferroni’s post hoc test were used to compare ACP and nanoZn composition. NanoZn(St) composition after storage was also compared with respect to the fresh nanoZn sample. When *p*-values are lower than 0.05, differences in the obtained numerical results were considered statistically significant: *(*p*-value < 0.05), **(*p*-value < 0.01), or ***(*p*-value < 0.001).

### 3.2 Stability of the Nanoparticles in Different Media of Practical Interest

#### 3.2.1 Long-Term Chemical and Structural Stability under Storage Conditions

The long-term chemical stability of the nanomaterials is a key aspect for their effective usage in agriculture. We found out that the composition of the nanoparticles, Ca, P, and Zn content, in water (10% w/v) remained almost constant after 2 years of storage at 4 °C (Table 1). In fact, the XRD pattern (Figure SI3) collected after this time displayed the characteristic broad band typical of the amorphous phase, confirming the long-term structural stability of nanoZn. On the other hand, control samples (ACP), without Zn and containing only citrate, converted into apatite after one month under the same conditions (Figure SI3). Hence, the long-term stability of nanoZn is due to the role of both ions, citrate and zinc, in the stabilization of the amorphous precursor. Previous studies demonstrated the individual role of citrate or zinc ions in stabilizing the metastable ACP phase,^10,41,42^ but this prominent effect due to the combinatorial role of citrate and zinc has never been previously observed, although ACP was synthesized in the presence of both ions.^42^

#### 3.2.2 Chemical Stability in Diluted Solutions: pH-Responsive Nutrient Release

The chemical stability of nanoZn was then studied under the conditions used during the experiments on tomato plants, i.e., aqueous media (pH = 7.4) at a concentration of 1000 ppm. After 24 h, we observed a low release of the ionic constituents, Ca^2+^, Zn^2+^, and PO_4_^3–^ ions, which is in agreement with the low water solubility of ACP.^38,43^ While 8.1 wt % of calcium and 6.6 wt % of phosphorus were released to the aqueous medium, only 0.1 wt % of zinc was quantified in water. This low release of Zn agreed with recent work reporting on a minimal Zn^2+^ release in water (0.4 ± 0.2 wt % at pH = 7) from zinc-doped amorphous calcium phosphate (Zn-ACP).^42^ The authors suggested that Zn^2+^ ions were withdrawn in the solid phase.

Citric acid is the most abundant carboxylic acid in tomatoes and the largest contributor to total titratable acidity. Citric acid concentration declines with progressing tomato maturation after ripening going from ca. 24 mM (green tomato) to 10 mM (ripe tomato).^31^ The pH of the fruit ranges from 4.2 to 4.6 during ripening.^31^ Thus, we have also evaluated the release of nutrients in citric acid (10 mM, pH = 4.5), with the aim of mimicking the conditions inside the tomato fruit to better understand the results obtained on tomato mineral nutrition. Interestingly, the nanoparticles completely dissolved after 24 h, releasing 100% of all its constituents (Table 2). Previous work also demonstrated higher Zn^2+^ release from Zn-ACP at acidic pH, attributing this effect to the higher solubility of ACP at an acidic pH value that enhances the release of Zn ions, as previously observed.^42,44^ Zinc delivery from Zn-ACP nanoparticles (333333 ppm) dispersed in lactate buffer at pH = 4.5 was close to 1 wt %,^42^ while Zn^2+^ were completely released from Zn-ACP microspheres at pH 2 and 4 (adjusted with diluted hydrochloric acid), using an initial concentration of 1000 ppm of nanoparticles,^44^ the same concentration that we used in our study.

**Table 2:** Relative Release of Ca, P, and Z from NanoZn in Different Media and pHs, Mimicking the Conditions in the Tomato Fruit^a^

		relative release		
media	pH_0_	Ca (wt %)	P (wt %)	Zn (wt %)
water	7.4	8.07 ± 0.46	6.55 ± 0.21	0.14 ± 0.02
citric acid	4.5	104.28 ± 4.35***	106.70 ± 3.86***	100.59 ± 3.29***

a Statistical analyses using the *t*-Student unpaired test were used to compare water and citric acid release. ***(*p*-value < 0.001).

Therefore, the complete dissolution of nanoZn observed in our study could be associated with the relatively low initial nanoparticle concentration (1000 ppm) compared to a previous study (333333 ppm),^42^ the acidic pH, and the effect of the carboxylic acid. Citrate molecules can play a dual role: (i) complexing the surface metal ions (e.g., Ca^2+^ and/or Zn^2+^) promoting their eventual detachment from mineral surfaces and (ii) forming complex with metal ions already released to the solution, thus decreasing the effective concentration of metals in solution, reducing the eventual saturation.^45^

### 3.3 Efficiency of the Nanoparticles in Plants: Nutrient Uptake in Tomato Plants

**Figure 3:**
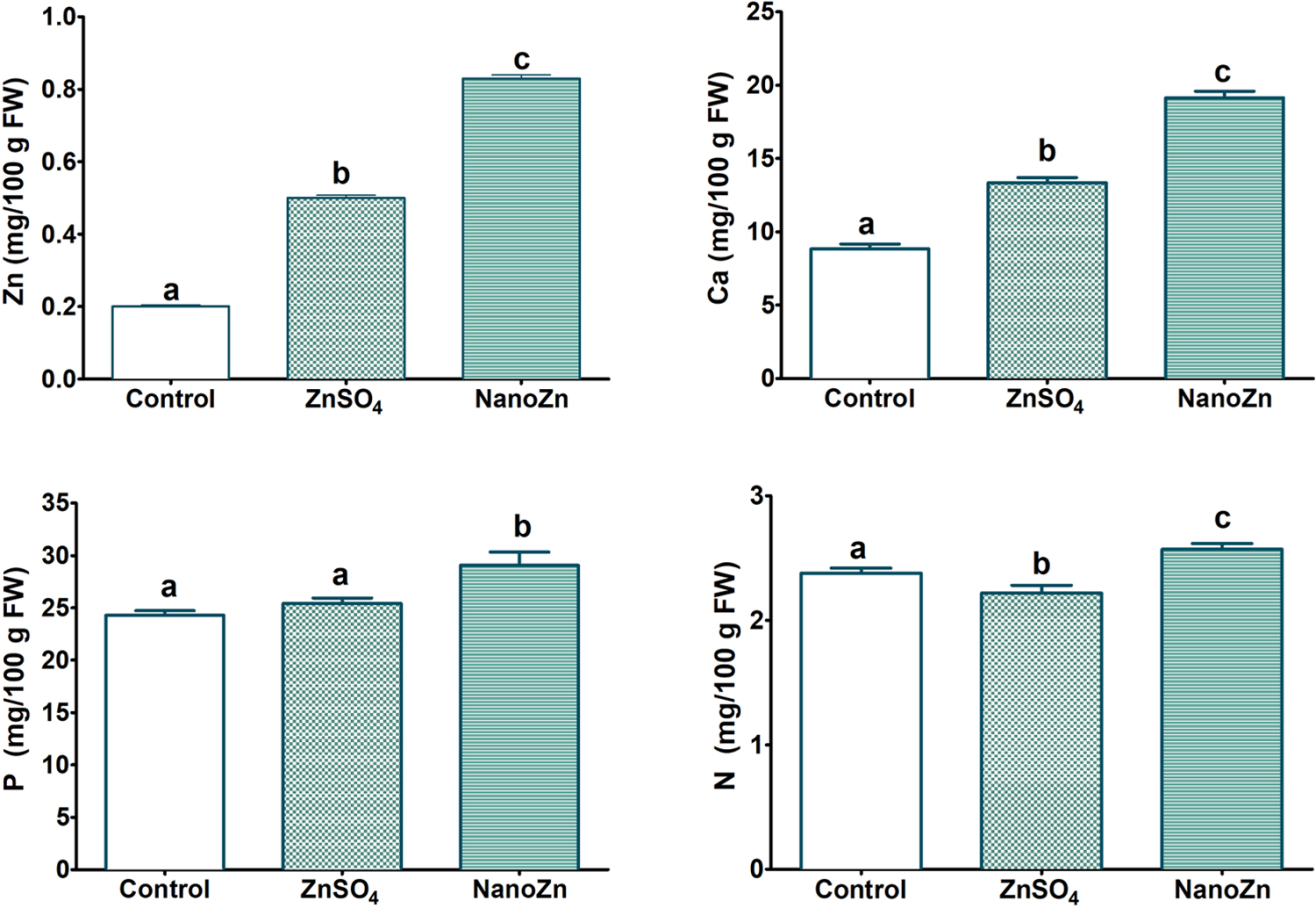
Average values (mg/100 g of FW) of element composition in the biofortified tomato fruit under Zn treatments and the control treatment. All parameters are given as averages with the corresponding standard errors. Letters indicate statistically significant differences (using one-way ANOVA) between treatments (*p* < 0.05).

The efficacy of nanotreatment was compared with respect to conventional zinc sulfate foliar application at the same Zn concentration (100 ppm). NanoZn produced a noticeable increase of the Zn content in tomatoes, exceeding by 352% the control and by 65% the tomatoes treated with ZnSO_4_ (Figure 3). The nanoparticles produced tomatoes supplying (per 100 g) up to the 7.82% (male) and 10.75% (female) of the daily recommended dietary allowance (RDA) of Zn for adults,^46^ values much higher than those obtained with the conventional or control treatments (Table SI2).

Previous studies demonstrated the higher Zn nutrient efficiency of zinc oxide nanoparticles (ZnO-NPs) with respect to conventional nanofertilizers (ZnSO_4_).^6,20^ Field experiments conducted by Zhang et al. demonstrated that the foliar application of ZnO-NPs (2 g/L) increased the Zn concentration of winter wheat grain to a greater extent than the application of ZnSO_4_ (7 g/L), exceeding by 47.6% control and 19.2% ZnSO_4_ treatment.^19^ Another study carried out during wheat germination in pot experiments revealed that soil treatment with ZnO-NPs or with ZnSO_4_ at 10, 20, 50, 100, 200, and 1000 mg/kg increased the Zn accumulation in different tissues of wheat seedlings.^21^ ZnO-NPs were more effective than ZnSO_4_ at increasing grain Zn content, exceeding by 230% control and 36.6% ZnSO_4_.^21^ However, variable results between experiments and crops have been reported for this type of nanoparticle.^8^

More interestingly, nanoZn treatment also increased the Ca, P, and N content in tomato fruits (Figure 3). The Ca and P content of the tomato treated with ZnSO_4_ increased compared to the control but to a much lesser extent. It is known that Zn favors the absorption of other important nutrients and specifically nitrogen, which is responsible for protein biosynthesis.^47^ However, the increase in N content was observed only when the tomatoes were treated with nanoZn (Figure 3).

**Figure 4:**
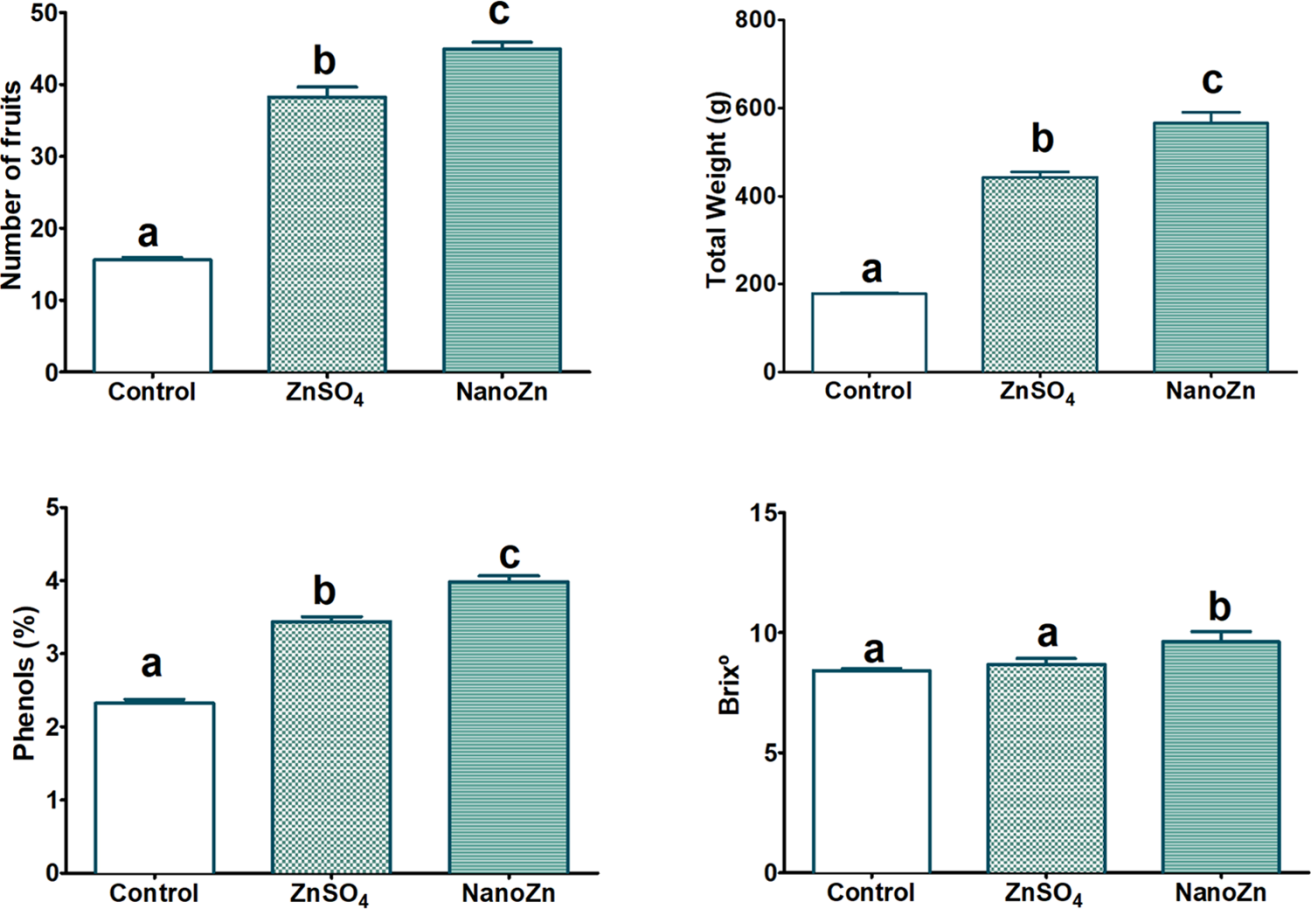
Average values of yield parameters of the tomato fruits. All parameters are given as average of 15 plants with the corresponding standard errors as error bars. Letters indicate statistically significant differences between treatments (*p* < 0.05).

**Figure 5:**
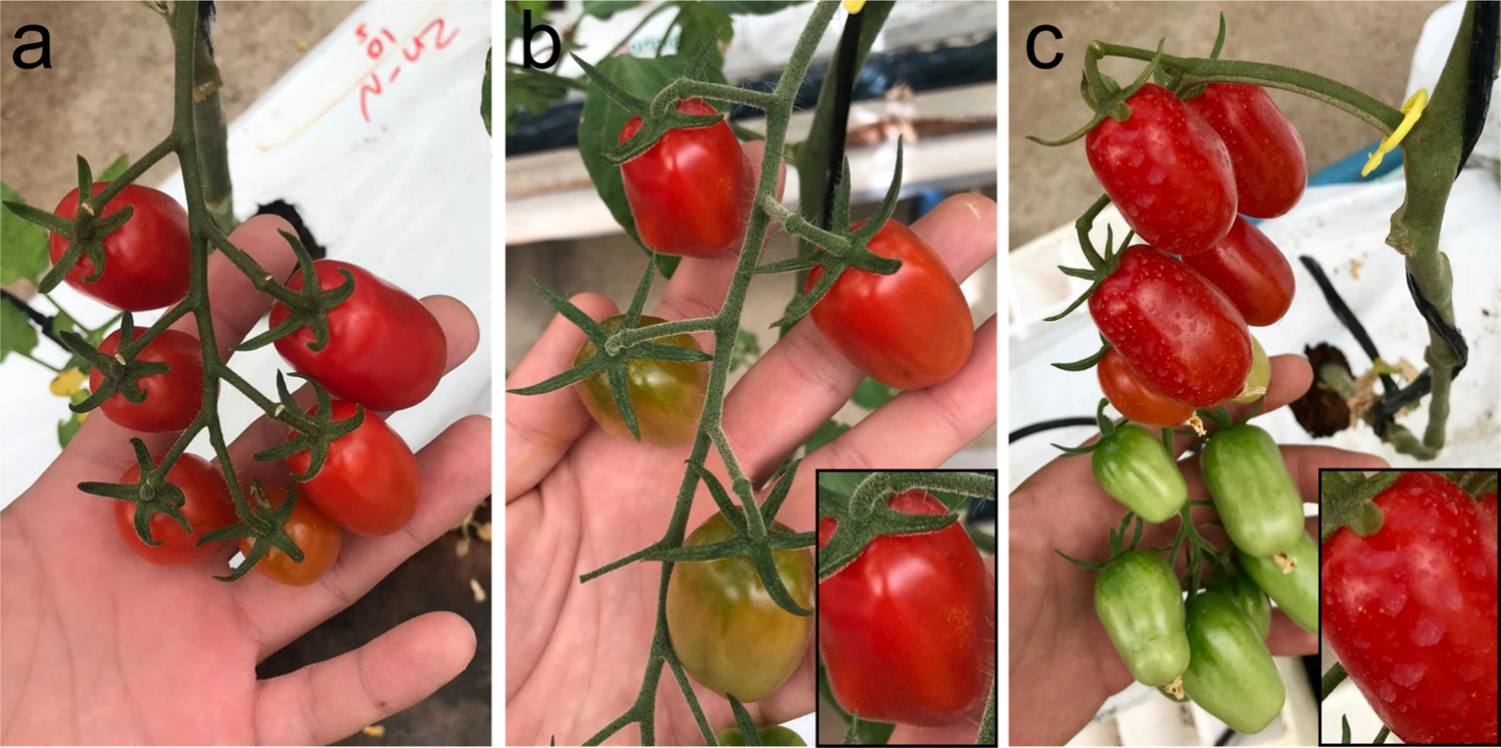
Representative pictures of the development of tomato fruits from (a) control plants and treated with (b) ZnSO_4_ and (c) nanoZn. Insets show an enlarged view of the selected tomatoes.

Another important observation was regarding the yields of the fruits. Tomato plants exposed to the foliar application of nanoZn nanoparticles produced the highest number of fruits and fruit total weight with respect to ZnSO_4_ and the control (Figures 4 and 5). Important parameters such as phenol content and °Brix were also increased with the nanoparticles in comparison to those of the conventional treatment (Figure 4). Zn can activate many enzymes involved in various biochemical pathways such as carbohydrate, protein, and growth regulator metabolism and thus promote the growth of crops.^5,16,48^ The increased fruit production obtained with the foliar application of nanoZn can be directly associated with the increase of N, P, and Ca contents, since these important plant macronutrients stimulate plant growth and crop production.^49^ The enhancement of plant growth and fruit production for Zn nanoformulations (ZnO-NPs) compared to soluble ZnSO_4_ have also been previously demonstrated in wheat, coffee plants, habanero peppers, and tomatoes, among others.^6,48^

### 3.4 Foliar Application of Nanoparticles: Mechanism of Absorption, Translocation, and pH-Responsive Delivery of Nutrients

The retention of nutrients on leaves can be prolonged with hydrophobic nanoparticles. The presence of –CH_2_ moieties of citrate on the nanoZn surface, as confirmed by the FTIR spectrum (Figure SI2b), enhances nanoparticle hydrophobicity^50^ and consequently the retention on the leaves. In fact, pictures of tomato plants revealed white spots on the tomatoes (Figure 5) and the surface of the leaves (Figure SI4) after nanoZn treatment, not observed on the tomatoes/leaves treated with ZnSO_4_. This prolonged retention was already observed on the leaves of vineyards treated with calcium phosphate nanoparticles.^51^

In addition, Zn^2+^ ions from highly soluble salts may have difficulty penetrating the lipophilic cuticle, thus limiting its bioavailability and reducing its nutrient use efficiency.^52^ Zn enters the plant (leaf apoplast) directly through stomatal pores, increasing Zn concentration in phloem tissue of the leaf from where it can be directly translocated to growing sinks (i.e., foliage, grain, and fruit).^53^ Zn transportation in the phloem is additionally limited to the binding to a chelator, such as nicotianamine (NA) or cysteine, which prevents its precipitation in the slightly alkaline phloem sap.^54^

Foliar-sprayed nanoparticles enter the leaves through stomata and are then translocated, via apoplastic and symplastic pathways, to different parts of the plant (including the fruit).^55^ NanoZn are stable in neutral or slightly alkaline media (Table 2), avoiding the delivery of Zn^2+^ and the subsequent precipitation and, consequently, favoring the Zn translocation inside the plant. Conversely, the high concentration of citric acid (10–24 mM, depending on the maturation state) and the acidic pH (4.2–4.6) of the tomato fruit trigger nanoparticle dissolution. Our data revealed that nanoZn were completely dissolved releasing 100% of their constituents (Table 2) in citric acid solution (10 mM, pH 4.5), mimicking the sap composition of the tomato fruit.^31^ NanoZn dissolution and the respective ionic release may explain the higher Ca, P, and Zn content of the tomato fruit treated with nanoZn, compared to control (water treatment) and zinc sulfate treatments (Figure 3). Although this is a plausible mechanism based on nanoparticle pH-dependent solubility and enhanced retention on the leaves, specific studies are needed to elucidate the complex mechanism involving nanoZn translocation and stability inside the plants. Based on the experimental design, the absorption of the nutrients took place before the harvest of the fruits, and the removal of the remaining product on the surface of the tomato during the washing procedure before nutrient analysis did not hinder the nutrient absorption.

### 3.5 Antimicrobial Activity of NanoZn: Protection against *P. syringae*

**Figure 6:**
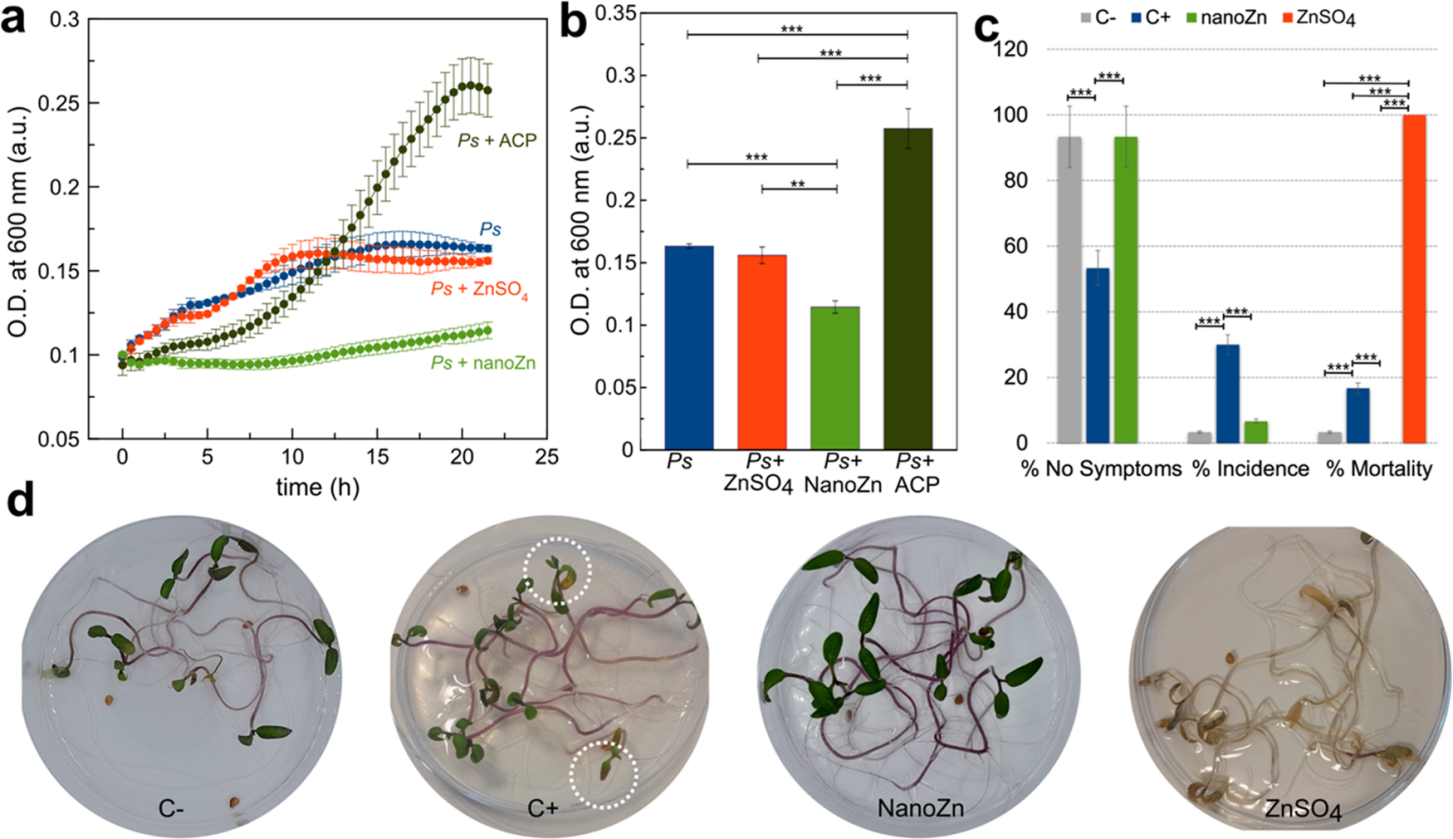
Antibacterial activity of nanoZn. (a) Growth curves of *Ps* in KB media and in the presence of ZnSO_4_, ACP, and nanoZn. (b) Optical density (OD) at 600 nm at the end of the growth experiments (time = 21 h) of panel a with the corresponding statistical analysis. (c) Percentage of seedlings showing *Ps*-related no symptoms, incidence, and mortality under each treatment: negative control C– (no inoculated + sterile water), positive control C+ (inoculated + sterile water), inoculated treated with nanoZn (100 ppm Zn), and inoculated treated with ZnSO_4_ (100 ppm Zn). (d) Photographs of tomato seedlings 12 days post inoculation with *Ps*. Dotted circles indicate leaves with chlorosis associated with *Ps* infection. Both statistical analyses in (b) and (c) were performed using the one-way ANOVA test and Bonferroni’s post hoc test. *p-value < 0.05, **p-value < 0.01, or ***p-value < 0.001.

Other interesting property of nanoZn was the inhibitory activity against *P. syringae* (*Ps*), the main cause of tomato bacterial speck, a disease that causes severe losses in tomato yield and quality worldwide, occurring particularly during cold and wet springs.^27^ We monitored the growth of the bacteria (measuring the optical density at 600 nm) in KB media at pH 4.5, starting from an optical density of 0.1 (Figure 6a,b). Under these conditions, the addition of ZnSO_4_ (100 ppm of Zn) did not inhibit the growth of the bacteria with respect to the control (Figure 6b), while the addition of the control nanoparticles, ACP, accelerated the bacterial proliferation (Figure 6a,b). This is likely due to the increase of pH associated with the release of species from the nanoparticles. *Ps* growth is favored at higher pHs as can be observed in Figure SI5. Interestingly, the addition of nanoZn to the media produced a strong inhibition of bacterial growth (Figure 6b), with the optical density being practically constant for 24 h (Figure 6a). Under these latter conditions, an increase of pH is also expected due to the release of ionic species, which might accelerate bacterial proliferation. However, nanoZn inhibit the proliferation even in media favorable to *Ps* growth.

The inhibitory effect of nanoZn was also evaluated in tomato seeds previously inoculated with *Ps* (Figure 6c,d). Bacterial virulence was evaluated at 12 days post inoculation (dpi). Noninoculated seedlings treated with sterilized water (C, Figure 6d) exhibited pigmented hypocotyl and healthy cotyledons. Seedlings exposed to *Ps* and treated with sterilized water (C+, Figure 6d) exhibited severe necrotic symptoms and extensive chlorosis, which is a hallmark of bacterial speck disease in foliar tissue (marked with dotted circles in Figure 6d).^37^ In this condition, seedlings showed 30% incidence and 17% mortality due to *Ps* infection (C+, Figure 6c). Surprisingly, we found that the application of nanoZn significantly reduced disease symptoms in tomato seedlings, which showed extremely healthy cotyledons and hypocotyl and null mortality (Figure 6c,d). On the other hand, the application of 100 ppm of Zn as ZnSO_4_ prompted to 100% of dead seedlings due to the phytotoxicity of soluble zinc fertilizers (Figure 6c,d).^6^ In this condition, to distinguish *Ps* symptoms from ZnSO_4_ phytotoxicity was quite difficult and *Ps* incidence was ruled out and considered null. There results confirm the important protective actions of nanoZn against *P. syringae* pv tomato infection.

### 3.6 Environmental Significance

Among the most interesting benefits of calcium phosphate nanoparticles, emulating the bone mineral, are their nontoxicity, biocompatibility, and biodegradability.^56^ Owing to these properties, calcium phosphate nanoparticles have been widely used in cosmetic (e.g., solar filters or toothpaste),^57^ drug delivery, and bone regeneration.^56^ Thus, considering that the nanoparticles are mainly composed of calcium, phosphorus, oxygen, and Zn, risks in the regulatory affairs are not expected.

Scaling up and economic aspects must be considered when designing a new (nano)technology for more sustainable agricultural practices. The synthesis of nanoZn has been scaled-up by increasing the volume of reaction from 200 mL (laboratory scale) to 50 L (pilot scale), which allowed increasing 250 times the production per batch (i.e., from 1.6 to 400 g per batch). Likewise, we proved that the engineered nanoparticles can be also produced by using low-cost technical-grade reagents and tap water (Section S2 of the Supporting Information), significantly lowering the production costs, without varying the properties of the particles (i.e., structure, morphology, or chemical composition, Figure SI6). The great advantage of the usage of impure but cheaper reagents produces a 35x price reduction in comparison to the use of analytical-grade reagents supplied by conventional chemicals vendors. Although the costs of the marketed zinc suppliers (e.g., zinc sulfate) are much lower, losses due to the high solubility and low retention of the conventional fertilizer, and the consequent environmental costs, should be considered.

The analysis of the environmental impacts of the engineered nanomaterials through their whole life, from the production to the application in the field, was also conducted. These engineered nanomaterials have multiple sustainability aspects. As for the sustainability of the production, the synthesis is carried out by a simple precipitation method based on the mixture of aqueous salt solutions, thus avoiding the use of hazardous solvents, and under mild conditions (neutral pH, room temperature, and atmospheric pressure). Regarding the environmental impact of Zn application in the fields, a special care of zinc sulfate management is highly recommended and losses to the environment should be strictly reduced. Zinc sulfate fertilizers are highly soluble in water (57 g/100 mL at 25 °C) releasing 100% of Zn when it enters in contact with water at a Zn dosage of 100 ppm. The high solubility of this conventional fertilizer prompted to high lixiviation rates and the need of repeated applications to the leaves during the growing cycle, which prompted serious environmental impact such as phytotoxic effect on the plants reducing the crop yield and quality and inhibitory activity of the growth and metabolism of soil microorganisms.^6,58^ Compared to highly soluble zinc sulfate, Zn incorporation into ACP nanoparticles slowed the Zn release in the neutral aqueous medium due to the intrinsic low solubility in water of calcium phosphate, only 0.14 wt % of Zn being released after 24 h in water (Table 2). However, the release of ions is favored in acidic media, as that inside the tomato. This pH-responsive release of ions makes the process more efficient, reducing the environmental impact of the usage of conventional zinc sulfate fertilizers.

The observed plant protection effect was another additional environmental significance associated with these multifunctional nanomaterials. NanoZn can be considered as an alternative to control plant diseases caused by *Ps*, the main cause of bacterial speck in tomatoes, which has a relevant impact in crop production worldwide. This would avoid (or significantly reduce) the use of environmentally harmful conventional strategies consisting in the spray of bactericides, such as copper compounds (e.g., copper hydroxide) with or without combination of fungicides or other pest-control chemicals,^59^ their application currently being under regulation (e.g., a maximum limit of 4 kg ha^–1^ year^–1^ of copper per application has been imposed in Europe).^27^ Therefore, the strategy reported here presents dual agricultural applicability, increasing crop yields and plant nutritional values and simultaneously avoiding the development of significant bacterial-based diseases. Therefore, the multifunctionality of these innovative nanomaterials makes in turn their usage more efficient and sustainable.

## Supporting Information Available

Details of analytical methods validation (Section S1); scale-up and cost reduction of nanoZn synthesis (Section S2); additional material characterization (Figures S1–S3); additional photograph of plant leaves after the treatments (Figure SI4); additional information on *Ps* growth (Figure SI5); characterization of low-cost nanoparticles (Figure SI6); conditions of bacterial growth and RDA of zinc, respectively (Tables SI1 and SI2) (PDF)
