# Supplementary material for: Multifunctional Nanomaterials for Biofortification and Protection of Tomato Plants
Source: Environ Sci Technol. 2023 Sep 27;57(40):14950–60. doi: 10.1021/acs.est.3c02559 (PMC10569043; doi:10.1021/acs.est.3c02559)
Supplement: Supplementary file 1 — es3c02559_si_001.pdf [file es3c02559_si_001.pdf]

Supplementary information for

Multifunctional Nanomaterials for Biofortification and Protection of Tomato Plants

Belén Parra-Torrejón.[a] *Andrés Cáceres,*[a] *Manu Sánchez,*[a,b] *Luis Sainz,*[c] *Miguel Guzmán,*[c] *Francisco J. Bermúdez-Perez,*[d] *Gloria B. Ramírez-Rodríguez,*[a,*] *José M. Delgado-López,*[a,*]

[a] Department of Inorganic Chemistry, Faculty of Science, University of Granada, Av. de Fuente Nueva, s/n, 18071 Granada (Spain).

[b] Institute of Nanoscience and Materials of Aragon, INMA-CSIC, C/ Mariano Esquillor, s/n, 50018 Zaragoza (Spain).

[c] Department of Agronomy, University of Almeria (RNM 151 PAIDI-UAL, ceiA3, CIAMBITAL), Carr. Sacramento, s/n, 04120 La Cañada, Almería (Spain).

[d] Nanointec S.L. Pol. Ind. de la Celulosa, C. Regaliz, 6, 04007 Almería (Spain).

Corresponding authors: J.M.D.-L. (jmdl@ugr.es), G.B.R.R. (gloria@ugr.es)

Table of Contents:

S1: Analytical methods validation……………………………………………...S2

S2: Scale-up and cost reduction……………………………………………...S3

Figure SI1…………………………………………………………………...S4

Figure SI2………………………………………………………………......S4

Figure SI3………………………………………………………......……S5

Figure SI4……………………………………………......……………S5

Figure SI5……………………………………………......………………S6

Figure SI6……………………………………………......………………S6

Table SI1……………………………………………......………………S7

Table SI2……………………………………......………………………S7

S1: Analytical methods validation

The quality assurance (QA) and quality control (QC) operations performed to validate the analytical instruments are described below:

The chemical composition and stability of nanoZn was analysed by inductively coupled plasma optical emission spectrometry (ICP-OES, PerkinElmer® Optima 8300) from Centre for Scientific Instrumentation of University of Granada (https://cic.ugr.es). PerkinElmer engineers carried out the installation qualification (IQ), operational qualification (OQ) and performance qualification (PQ) of the equipment. Moreover, they trained to qualified staff for the periodic equipment maintenance, including the replacement of consumables as torches, injectors or

nebulizers, and the verification of the its correct performance (*i.e.,* automatic detector calibration, alignment of the torch and UV/Vis Wavelength calibration checked with manganese standard).

The trained technician carried out the precision test and background equivalent concentration (BEC) test before each measurement. For the precision test, manganese standard (10 $mg \cdot L^{-1}$) at wavelength of 257.610 nm is used. The Relative Standard Deviation (RSD) is calculated from 10 measurements, being RSD value lower than 2.5% suitable to pass the test. BEC Test compared the intensity of a blank to the value of Mn standard. BEC value must be less or equal to 0.04 $mg \cdot L^{-1}$ to pass the test.

The calibration curve for Ca, P and Zn quantification was carried out in the range of 5-50 mgL^{-1} using a blank (ultrapure MilliQ water) and four standard solutions prepared by diluting Ca standard (1000 $mg \cdot L^{-1}$, PerkinElmer Pure, CAS# 7440-70-2), P standard (1000 $mg \cdot L^{-1}$, PerkinElmer Pure, CAS# 7632-18-5) and Zn standard (1000 $mg \cdot L^{-1}$, PerkinElmer Pure, CAS# 7440-66-6). Blank samples are also measured between standards and nanoZn samples to validate the cleaning of the operating tubes during sample exchange.

S2: Scale-up and cost reduction

NanoZn was synthesized with technical grade (TG) reagents and tap water to reduce the cost of the production. The technical grade reagents were purchased from Vadequimica (Spain) with the following purity: calcium chloride dihydrate ($CaCl_2 \cdot 2H_2O$, 77.0%), potassium citrate tribasic monohydrate ($K_3(C_6H_5O_7) \cdot H_2O$, 99.0%), zinc chloride ($ZnCl_2$, 98%), potassium phosphate dibasic anhydrous (K_2HPO_4, 98.0%) and potassium hydroxide (KOH, 90%). We evaluated the feasibility of carrying out the same procedure described in section "Synthesis and characterization of nanoZn" (page 7) by using technical grade reagents and scaling-up the

reaction volume from 200 mL (laboratory scale) to 50 L (pilot scale). Then, the sample were characterized by XRD, FTIR and TEM (Figure SI7).

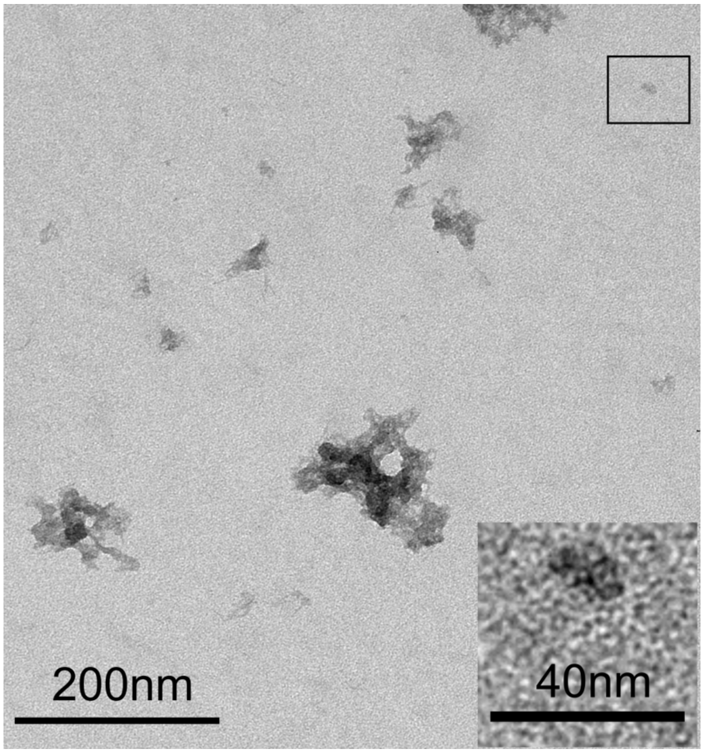

Figure SI1. TEM micrograph of amorphous calcium phosphate nanoparticles (ACP, control).

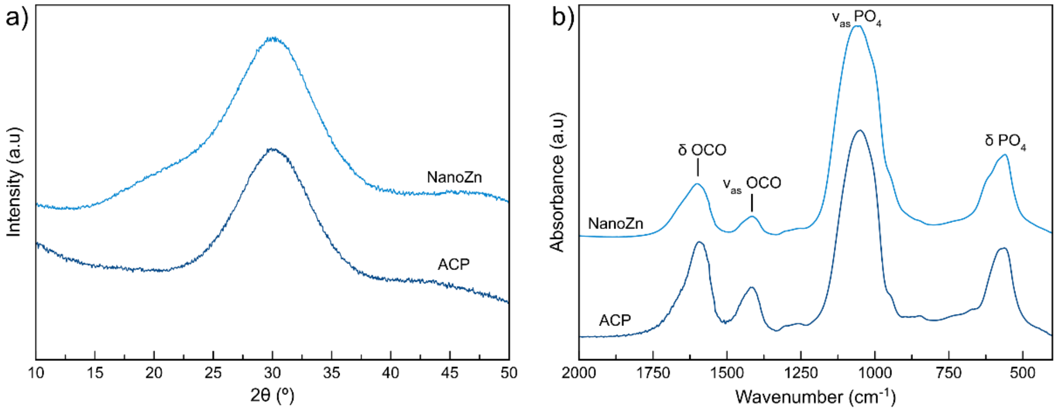

Figure SI2. (a) XRD patterns and (b) FTIR spectra of nanoZn and ACP nanoparticles (control).

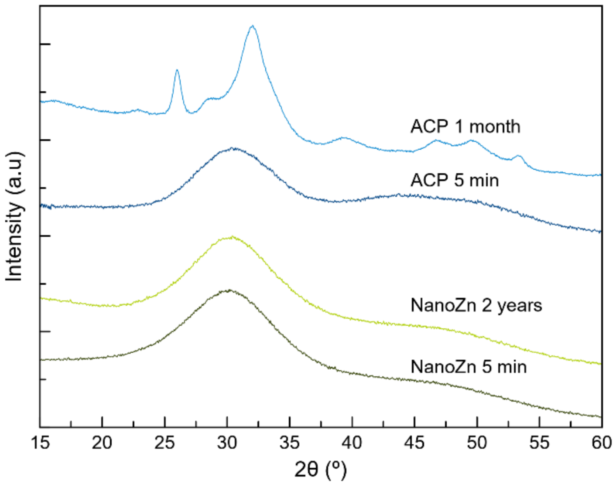

Figure SI3. XRD patterns of control (ACP) and NanoZn nanoparticles stored in aqueous media at 4 ºC. ACP transformed to nanocrystalline apatite after 1 month of storage whereas NanoZn remained as amorphous phase after 2 years.

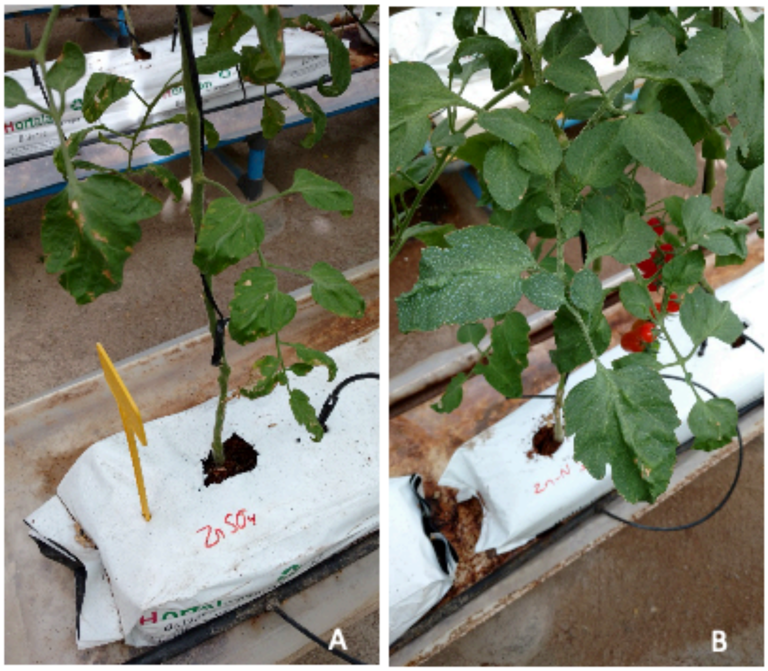

Figure SI4. Representative picture of tomato plants after foliar application of $ZnSO_4$ (A) and NanoZn (B). White spots of nanoZn can be clearly observed on the leaves.

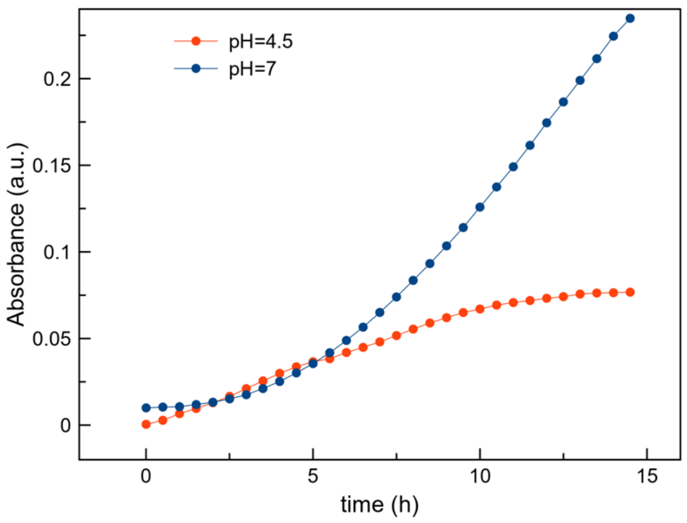

Figure SI5. Growth curves of *Ps* collected at 600 nm at different pHs, demonstrating the impact of the pH in the bacteria proliferation.

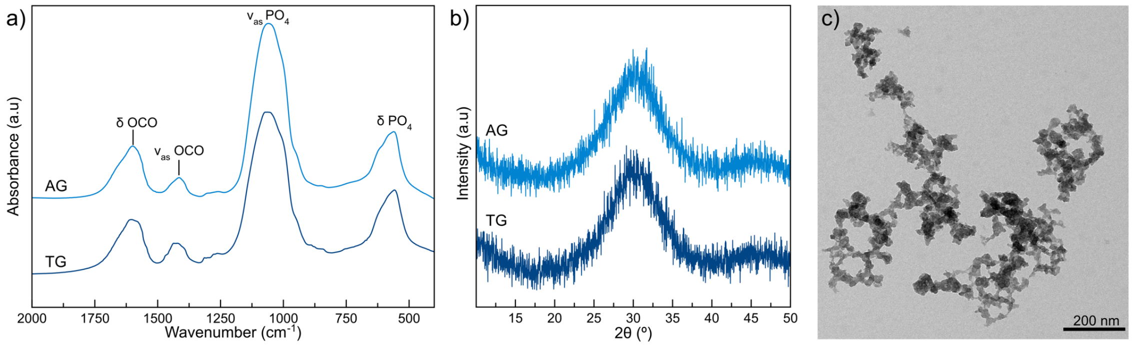

Figure SI6. FTIR spectra (a) and XRD patterns (b) of nanoZn synthesized with analytical grade reagents at the laboratory (200 mL, AG) and technical grade reagents at high volume (50 L, TG). C) TEM micrograph of nanoZn nanoparticles synthesized through the scaled-up process (50 L, TG).

Table SI1. Summary of the conditions used to study the growth inhibition of *Ps*.

Treatments	Conditions	Zn concentration (ppm)
Ps	20 μl bacterial suspension + 80 μl KB + 100 μl H_2O	0
Ps + $ZnSO_4$	20 μl bacterial suspension + 80 μl KB + 100 μl $ZnSO_4$ in H_2O	100
Ps + nanoZn	20 μl bacterial suspension + 180 μl of nanoZn in KB/H_2O (1:1 v/v)	100
Ps + ACP	20 μl bacterial suspension + 180 μl of ACP in KB/H_2O (1:1 v/v)	0

Table SI2. Daily intake and corresponding coverage of RDA for Zn through the consumption of portions (100 g) of tomato per day, fortified through different treatments ($ZnSO_4$ and nanoZn) and non-fortified (control), by adult's male and female.

Treatment	Daily Zn Intake (mg Zn/Day)	RDA-Zn coverage (%)	
		Male	Female
Control	0.200±0.005 a	1.73	2.38
Zn-SO_4	0.500±0.008 b	4.73	6.50
NanoZn	0.830±0.010 c	7.82	10.75
